# KASSPer: kinase active site structure prediction using protein and ligand language models and its application to virtual screening

**DOI:** 10.1093/bioinformatics/btag481

**Published:** 2026-07-01

**Authors:** Wonkyeong Jang, Woong-Hee Shin

**Affiliations:** Department of Biomedical Informatics, Korea University College of Medicine, 161 Jeongneung-ro, Seongbuk-Gu, Seoul, 02708, Republic of Korea; Department of Biomedical Informatics, Korea University College of Medicine, 161 Jeongneung-ro, Seongbuk-Gu, Seoul, 02708, Republic of Korea; Molecular Design Team, Arontier Co., 241 Gangnamdae-ro, Seocho-Gu, Seoul, 06735, Republic of Korea

## Abstract

**Motivation:**

Structure-based virtual screening (SBVS) is limited by the rigid-receptor assumption, which is particularly problematic for kinases that adopt multiple active-site conformations but are experimentally biased toward a single state. Although ensemble screening can address this limitation, it remains computationally expensive.

**Results:**

We introduce KASSPer (Kinase Active Site Structure Predictor), a framework that predicts kinase active-site conformational states using protein and compound language models. Given a kinase amino acid sequence and a ligand SMILES string, KASSPer enables ligand-specific conformer selection prior to SBVS, potentially reducing the computational cost associated with exhaustive ensemble screening. Benchmarking on the DUD-E kinase subset demonstrates that KASSPer-guided screening outperforms the tested ensemble-based approach across the evaluation metrics.

**Availability and Implementation:**

The implementation for model loading and inference is available at the GitHub repository https://github.com/kucm-lsbi/KASSPer.

## Graphical Abstract

**Figure:**
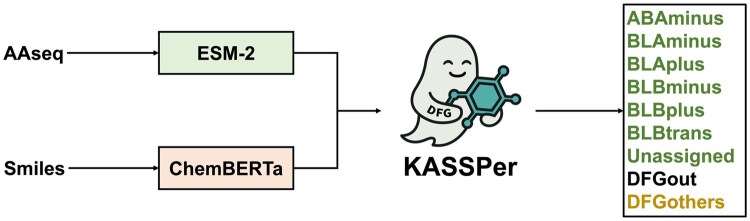

## 1 Introduction

Structure-based virtual screening (SBVS) has become an indispensable part of drug discovery by utilizing the three-dimensional structural information of target proteins to predict protein–ligand binding poses and corresponding affinities to find promising hit candidates from a chemical library. Despite its proven utility, SBVS often struggles to account for receptor conformational flexibility. Structural states and binding-site geometries can vary substantially during ligand binding (Bordogna *et al.* 2011, Fan *et al*. 2009). This limitation can lead to false positives when screening against a single static structure, thereby reducing hit rates and downstream success in hit identification.

To address this challenge, ensemble screening employs multiple receptor conformations derived from X-ray crystallography, NMR ensembles, or molecular dynamics simulations to capture the conformational space of the target diversely (Modi and Dunbrack 2019). In this approach, a given ligand is docked to multiple receptor structures. A representative score is then selected for compound ranking. While ensemble screening can improve the finding of hit molecules from the library, it comes at the expense of substantial computational cost, as each ligand must be docked across an entire set of receptor structures. Furthermore, selecting representative conformations from a large pool remains nontrivial and may introduce bias if the structural ensemble is not sufficiently diverse or balanced.

Protein kinases represent one of the most extensively studied and therapeutically relevant target families, with over 500 members regulating key cellular processes. Kinase active sites are characterized by the conformations of the DFG motif and the αC-helix. These structural features give rise to well-defined binding modes, including DFGin, DFGout, and intermediate states (Berman *et al.* 2000). Tools like KinCoRe classify kinase conformations by analysing activation-loop positions, αC-helix orientation, and DFG dihedral angles (Modi and Dunbrack 2022). However, the Protein Data Bank (PDB) remains heavily biased toward the DFGin BLAminus state, limiting the discovery of inhibitors that preferentially bind less-populated or transient kinase conformations.

Recent advances in the protein structure prediction field, such as AlphaFold and its derivatives (Abramson *et al.* 2024, Krishna *et al.* 2024, Passaro *et al.* 2025), have demonstrated remarkable improvements in accuracy and have been applied to generate diverse conformations for ensemble screening workflows (Song *et al.* 2024). Despite these advancements, docking a molecule library against a large number of predicted models requires high computational demand and may yield redundant or non-physiological conformations.

In this study, we introduce KASSPer (Kinase Active Site Structure Predictor). This novel algorithm predicts the most probable kinase conformation adopted upon binding of a given ligand. The program utilizes the primary sequence of the kinase and ligand descriptors as inputs. To develop a predictive framework for kinase–ligand compatibility, a range of traditional machine learning algorithms and deep learning architectures were evaluated to ensure robust performance across various modeling approaches. In accordance with the benchmark study, a stacking ensemble combining LightGBM and Random Forest (RF) was selected as the predictor. By predicting a receptor conformation tailored to each query compound, KASSPer restructures the virtual screening pipeline. Instead of exhaustive ensemble screening, KASSPer adopts a targeted strategy in which each compound is docked to its predicted structure. We benchmarked this approach using the DUD-E (Mysinger *et al.* 2012) kinase subset. The study revealed substantial enhancements in enrichment factors at early retrieval rates, thereby underscoring its capacity to expedite the identification of conformation-selective kinase inhibitors. This strategy has the potential to enhance the efficacy of SBVS, facilitating the identification of hit compounds for various targets with structural diversity.

## 2 Methods

### 2.1 Benchmark dataset for kinase structure prediction and annotation

We extracted human kinase crystal structures from the KLIFS database (Kanev *et al.* 2021, accessed May 2025). The database contains unique complexes of kinase catalytic domain structures and their respective binders, extracted from PDB. In addition, it provides interaction information between the protein and the compound. Out of 6336 human crystal structures with bound ligand in KLIFS, 50 PDBs could not be accessed, resulting in 6286 structures. After splitting PDB structures into chains and removing redundant protein chain-ligand complexes, 7673 pairs remained. Subsequently, exact duplicates were removed based on protein sequences, ligand SMILES, and conformational labels. If identical protein–ligand pairs were associated with inconsistent labels, all related entries were excluded, resulting in a final dataset of 7353 pairs.

The conformational states of the benchmark set were annotated by Kinase Conformation Resource (KinCoRe) (Modi and Dunbrack 2022) standalone version. The binding site of kinases contains an activation loop that features the Asp-Phe-Gly (DFG) motif in the N-terminal region. This motif plays a crucial role in anchoring ATP to the active site. KinCoRe initially classifies kinase conformations based on the position of the activation loop. The three primary states are DFGin, DFGinter, and DFGout. In the DFGin state, Phe of the motif is located within the ATP-binding pocket, enabling the kinase to bind ATP. This configuration is designated as the active state. Conversely, DFGout conformation directs the Phe out of the ATP binding pocket, thus classifying it as an inactive state. The three primary classes are subdivided according to the backbone dihedral angles of X-DF and the χ1 angle of Phe, where X denotes a residue preceding the motif. In the system, the letters A, B, and L are assigned to alpha, beta, and left-handed, respectively. The Phe χ1 angle is designated as plus, minus, and trans for π/3, −π/3, and π, respectively. Consequently, the predominant class, designated as DFGin, comprises seven distinct subclasses: BLAminus, BLAplus, ABAminus, BLBminus, BLBplus, BLBtrans, and Unassigned. In contrast, DFGinter (comprising BABtrans and Unassigned) and DFGout (consisting of BBAminus and Unassigned) are characterized by a mere two subclasses. In the present study, since the sample size for subclasses is inadequate, the two subclasses of DFGout and DFGinter with Unassigned_Unassigned were aggregated and denoted as the 'DFGout’ and ‘DFGothers’ classes, respectively. Ultimately, nine structural states—ABAminus, BLAminus, BLAplus, BLBminus, BLBplus, BLBtrans, Unassigned, DFGothers, and DFGout—were designated as the prediction targets of the model.

The human kinase benchmark dataset was divided into three sets: a training set comprising 80% of the total data, a validation set (10%), and a test set (10%) using a ligand scaffold-aware split. Specifically, Murcko scaffolds were generated from ligand SMILES using RDKit (Landrum *et al.* 2025), and the resulting groups were assigned to each subset to satisfy the target split ratio while minimizing data leakage from structurally related ligands.

To validate the cross-species generalization, 396 mouse kinase structures were also extracted from the KLIFS database. After removing duplicated ligands, 286 unique protein-ligand pairs were obtained and constructed into the mouse benchmark set. The dataset was constructed using the same protocol as the human kinase. In contrast to human kinases, none of the structures were annotated as BLBtrans, resulting in eight states. The distribution of crystal structures for each class is shown in Fig. S1.

The sequence similarities between the human kinase test set and the mouse set were also analysed using MMSeqs2 (Version 17.b804f) to measure how similar they are. Pairwise sequence comparisons were conducted using the search module, with a sensitivity of 7.5, an *E*-value cutoff of 1 × 10^−5^, and coverage mode 2, with a minimum coverage threshold of 0.5. Also, the compound similarities were measured using extended-connected fingerprint (ECFP) 4-based Tanimoto similarity. The distribution of pairwise sequence and compound similarities is illustrated in Fig. S2.

### 2.2 Pre trained language models for kinase and compounds

To predict the conformational state of a kinase for a given compound, we utilized language models to embed molecules. To extract protein sequence embeddings, we used the ESM2 (Lin *et al.* 2023) protein language model. For each protein sequence, we utilized the output of the last hidden state of the ESM2 model. Only the hidden state values of the valid tokens corresponding to the actual protein sequence, excluding padding tokens, were converted to a fixed-size vector representation by mean pooling. The resulting 2560-dimensional embeddings were used for subsequent model training.

The SMILES sequence embeddings of the ligands were generated using the ChemBERTa (Chithrananda *et al.* 2020) model. ChemBERTa is based on the RoBERTa architecture and is a pre-trained model with a masked language modeling approach using the ZINC dataset. For each ligand’s SMILES sequence, the model’s last hidden state was extracted and converted into a fixed-size vector by performing mean pooling of the sequence tokens. The embedding with 768 dimensions was utilized as input for training.

### 2.3 Machine learning and deep learning models and hyperparameter optimization

#### 2.3.1 Machine learning models

In this study, we employed a variety of machine learning methods, including tree-based ensemble models, linear models, and support vector machines (SVMs) (Cortes and Vapnik 1995). For tree-based methods, XGBoost (Chen and Guestrin 2016), LightGBM (Ke *et al.* 2017), CatBoost (Prokhorenkova *et al.* 2018), and RF (Breiman 2001) were employed. For linear models, the implementation of logistic regression (Cox 1958) and SVM was undertaken. To optimize the hyperparameters of each model, the grid search (Pedregosa *et al.* 2011) was applied to identify the optimal combination. The hyperparameters are listed in the Table S1. All the machine learning models were utilized from scikit-learn.

#### 2.3.2 Deep learning models

Two architectures for utilizing deep learning were designed. The first model is concatenation. The embeddings of the protein and ligand were concatenated, and a multi-layer perceptron (MLP) (Rumelhart *et al.* 1986) was constructed to create an input vector. Subsequently, the vector traversed successive layers of hidden nodes, with a depth of 1024, 512, and 256 nodes, respectively. Each hidden layer implemented the rectified linear unit activation function (Nair and Hinton 2010), with a dropout rate of 0.3 applied to mitigate the risk of overfitting.

The second is a cross-attention (Vaswani *et al.* 2017) model that integrates protein and ligand embeddings. Each input embedding was projected to the same dimension through a linear transformation, and then information was exchanged between them through a bidirectional cross-attention mechanism. The two vectors obtained through the attention mechanism were combined and fed into an MLP for subsequent classification.

Both models were trained following the common protocol. Models were trained for 50 epochs with an initial learning rate of 1e−3. The learning rate was gradually decreased using a cosine annealing scheduler (Loshchilov and Hutter 2016). Adam (Kingma and Ba 2014) and AdamW (Loshchilov and Hutter 2017) were utilized as optimizers, with weight decay exploring values of 1e−6, 1e−5, and 1e−4, and the optimal model was identified based on the Matthews correlation coefficient (MCC) on the validation set. The concatenation and cross-attention architectures contain 4 067 337 and 4 335 625 trainable parameters, respectively.

#### 2.3.3 Stacking ensemble

To enhance the model’s performance and broaden its generalizability, we employed the stacking ensemble technique (Wolpert 1992), which integrates two models from the top three machine learning models: XGBoost, LightGBM, and RF. From each pre-trained base model, the predicted probabilities were organized into new features and provided as input to a meta-model using logistic regression. The meta-model was trained with the following parameters: multi_class='multinomial,' solver='lbfgs,' and max_iter = 200. Following the training process, the model was utilized to make final classification predictions.

#### 2.3.4 Embedding dimensionality reduction

To analyse the sensitivity of the models, the dimensionality of the protein embeddings was reduced, and the predictive performance of the models was evaluated to investigate whether they depend on high-dimensional representations. Principal component analysis (PCA) was employed to reduce the ESM2 embedding dimension. The original 2560-dimensional embeddings extracted from the final hidden layer of the ESM2 model were reduced to 128 dimensions using PCA implemented in scikit-learn. The PCA transformation was subsequently implemented on the embedding vectors derived from the training dataset. Subsequently, the learned PCA model was applied to transform all embeddings into a lower-dimensional representation. The PCA model that was fitted was also stored and reused during the evaluation process to ensure that the features were transformed consistently.

### 2.4 Classification model performance evaluation metrics

To comprehensively evaluate the performance of the classification model, the MCC was used as the primary metric, and precision, recall, and F1-score served as secondary metrics. All metrics are calculated based on the values of true positive (TP), false positive (FP), true negative (TN), and false negative (FN) in the confusion matrix. Precision is the ratio of TPs to all predicted positives (TP + FP), while recall is the percentage of TPs to all actual positives (TP + FN). F1-score is a harmonic mean of precision and recall that evaluates the balance between the two metrics. The MCC, a class imbalance robust metric, includes all elements of the confusion matrix, calculated as follows:


$$\mathrm{MCC}=\frac{\mathrm{TP}\times \mathrm{TN}-\mathrm{FP}\times \mathrm{FN}}{\sqrt{\left(\mathrm{TP}+\mathrm{FP})(\mathrm{TP}+\mathrm{FN})(\mathrm{TN}+\mathrm{FP})(\mathrm{TN}+\mathrm{FN}\right)}}$$

The value of MCC can range from +1 (perfect prediction) to -1 (perfect mismatch).

### 2.5 Applying KASSPer to SBVS and comparing with other methods

#### 2.5.1 Benchmark dataset

To ascertain the validity of the KASSPer prediction for drug screening, the DUD-E kinase subset, which consists of 26 kinases, was employed. Among them, SRC kinases were excluded from the evaluation since the reference crystal structure provided was not of human origin. Each kinase possesses a representative structure for SBVS, active compounds that were experimentally validated as binders to the target protein with affinities (IC50, EC50, Ki, and Kd) of 1 μM or better, and decoy compounds that do not bind to the receptor but are physico-chemically similar to the actives. From the ZINC database (Irwin and Shoichet 2005), the DUD-E protocol selected decoy molecules through a process of molecular matching. This process entailed the alignment of six physico-chemical properties–molecular weight, logP, number of rotatable bonds, number of hydrogen bond donors and acceptors, and net charge–with those of the active molecules. The selected compounds were then subjected to a subsequent ranking procedure, employing the Tanimoto coefficient to the active molecules using ECFP4. This approach identified the top 25% of dissimilar compounds for further consideration. The reduction of analogue bias was achieved through the aggregation of compounds according to their Bemis–Murcko atomic frameworks. The decoys were finally selected randomly to ensure 1:50 active-to-decoy ratio.

With the kinase subset, two benchmarks were performed: docking and screening. For docking, the co-crystallized compound was re-docked to the receptor in order to evaluate whether using KASSPer-predicted structures is helpful to reproduce the binding pose. KIT was excluded from the docking evaluation because the co-crystallized ligand structure had disordered functional groups. For screening, the active and decoy molecules provided in DUD-E set were docked to the KASSPer-predicted conformation and compared to the ensemble screening provided by Song *et al.*

#### 2.5.2 KASSPer, ensemble, and boltz-2 docking protocol

To get the diverse structure of kinases, the structures using AF2 with a multi-state modeling (MSM) protocol (Song *et al.* 2024) were employed. All human kinase experimental structures were retrieved from the KLIFS database and subsequently classified by KinCoRe to establish a template pool for each conformational state. The query kinase sequence was subjected to prediction by means of the MSM protocol, with the objective of generating a model encompassing all KinCoRe states for the designated query protein. For each structural state, the protocol identifies the top five templates with the highest sequence similarity to the query protein. In lieu of generating multiple sequence alignments, MSM employs a pairwise sequence alignment with the query protein and the single template structure, thereby biasing the generated model toward the kinase state represented by the template. Given that AF2 generates five models per single run, 25 models were predicted for each structural state. Subsequently, KinCoRe is utilized to ascertain that the predicted models exhibit the same structural state as the templates. Structures that did not align with the template’s structure were eliminated. Among the remaining models, the structure that demonstrates the highest pLDDT is selected for each state. For the modeled structures, the docking was performed using AutoDock-GPU (Santos-Martins *et al.* 2021). After aligning the model to the crystal structure, a docking box was defined as a cubic box with a side length of 22.5 Å. The center of the box was set to the geometrical center of co-crystallized ligand. AutoDock-GPU was executed with default parameters except for nrun = 50, the number of generated poses. For KASSPer screening, the kinase conformation was predicted for every single compound in the DUD-E set. Subsequently, the molecule was docked to the predicted conformation. In the event that KASSPer predicted a conformation as DFGout, docking was performed for DFGout_BBAminus and DFGout_Unassigned conformations. The result with the lower score was then used for screening. Similarly, in instances where the prediction was designated as DFGothers, the lowest docking score derived from the DFGinter_BABtrans, DFGinter_Unassigned, and Unassigned_Unassigned categories was employed for the screening process. For ensemble screening, the representative pose and score of a compound were selected based on the lowest AutoDock energy across all MSM models.

The docking benchmark was also compared with a recent complex co-folding method using deep learning. Boltz-2 (Passaro *et al.* 2025) was employed to predict the complex structures of the given kinase sequence and ligand SMILES string. The model was executed with six recycling steps, 200 sampling steps, and one diffusion sample.

#### 2.5.3 Evaluation metrics

The docking benchmark was evaluated by three metrics: the success rate of predicting conformational state of kinases, the root-mean-square-distance (RMSD) of predicted binding poses to the crystal structure, and the docking success, which is defined as RMSD ≤ 2 Å. Spyrmsd (Meli and Biggin 2020), which considers molecular symmetry to calculate RMSD, was utilized to evaluate the metric.

For the VS benchmark, active and decoy molecules were ranked according to the docking score. The area under the curve (AUC) and enrichment factors (EFs) were calculated to evaluate the screening performance. AUC is a metric that quantifies the discriminatory capabilities of a model in distinguishing between active and inactive compounds. AUC values approaching one are indicative of superior performance, and 0.5 corresponds to random retrieval.

EF is an indicator of the ability of an active compound to enrich within a certain percentage of the top docking scores compared to random selection. The enrichment factor at the top x% (EFx%) is calculated as follows:


$$EF_{x\%}=\frac{N_{\mathrm{actives},x\%}/N_{\mathrm{total},x\%}}{N_{\mathrm{actives}}/N_{\mathrm{total}}}$$

where Nactives, x% and Nactives are the number of active compounds and in the top x% and the library, respectively. Similarly, Ntotal, x% and Ntotal are the number of active compounds within the top x% and the entire dataset, respectively. In this study, the EF values at the top 1%, 5%, and 10% were calculated.

Furthermore, to quantify how effectively KASSPer distinguishes known binders from non-binders in terms of structural preferences, we calculated the Silhouette score using the 9-dimensional probability vectors output by KASSPer for each compound, as follows:


$$s(i)=\frac{b(i)-a(i)}{\max\left(a(i),b(i)\right)}$$

where *a*(*i*) is the mean intra-cluster distance to compounds in the same group (active or decoy) and *b*(*i*) is the mean nearest-cluster distance to compounds in the opposite group. The overall Silhouette score for each target was obtained by averaging *s*(*i*) across all active and decoy compounds. The overall Silhouette score ranges from −1 to +1. Positive values indicate separation of the conformational probability distributions between the two groups, near-zero values indicate overlap, and negative values indicate that the structural preferences of actives and decoys are not distinguished.

## 3 Results and discussion

### 3.1 Performance of KASSPer for predicting conformational state

The performance of the prediction models was validated on the human test set (735 structures) and mouse set (286 structures) to measure cross-species generalization. The human benchmark set was used as the primary evaluation, while the mouse set was employed to provide insight into generalization performance. The top three MCC models on both sets are presented in Table 1. A more detailed result can be found in the Table S2.

**Table 1:** MCC values of the top three single models, deep learning models, and stacking ensembles.

Test set	Single model					Stacking ensemble		
	XGBoost	LightGBM	RF	Cross Attention	Concatenation	XGBoost + Light GBM	LightGBM + RF	XGBoost + RF
Human	0.746	0.741	0.721	0.687	0.682	0.745	0.744	0.743
Mouse	0.524	0.506	0.487	0.446	0.394	0.509	0.530	0.533

In the human test set, XGBoost attained the highest MCC of 0.746, followed by LightGBM (0.741) and RF (0.721). On the mouse set, the top three models are the same as the human test set: XGBoost performed the highest performance with an MCC of 0.524, followed by LightGBM (0.506) and RF (0.487). To observe whether stacking ensembles could improve performance and robustness, the top three models were combined. Among the three combinations, the ensemble of XGBoost and LightGBM showed the highest MCC on human data with an MCC of 0.745. The XGBoost with RF ensemble demonstrated the most effective generalization on the mouse data with an MCC of 0.533.

**Figure 1:**
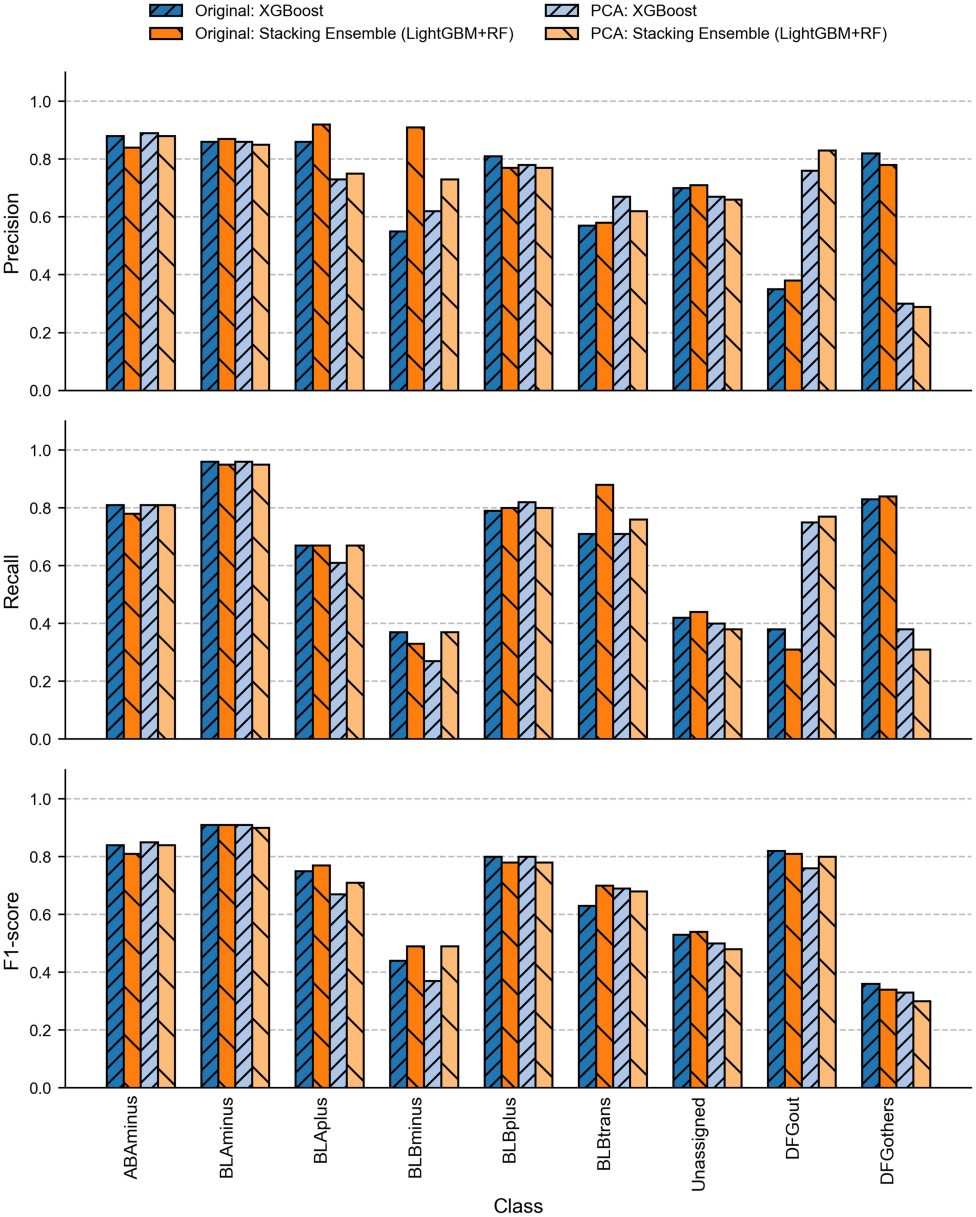
Comparison of class-wise prediction performance between XGBoost and a stacking model of LightGBM and RF, including models trained on PCA-transformed features. Performance is reported in terms of precision, recall, and F1-score for each class.

To examine the effect of training data size on model performance, learning-curve analyses were conducted for each model evaluated in this study (Fig. S3). As the training dataset increased, the performance of models on the test set showed a gradual improvement and reached a plateau. We also assessed the dependency of predictive performance on the high dimensionality of the language-model embeddings by applying PCA to reduce the protein embedding dimensionality before training. On the human test set, classification performance remained largely unchanged after dimensionality reduction (Fig. 1; Table S3). These findings suggest that a substantial portion of the predictive signal is retained within a lower-dimensional subspace.

Contrary to prevailing expectations, tree-based machine learning models exhibited superior performance in comparison to more complex deep learning frameworks: cross-attention and concatenation. These relatively sophisticated models yielded MCC values of 0.687 and 0.682 on the human set, respectively. In addition, their performance further diminished to 0.446 and 0.394 on the mouse set. Recent studies have indicated that these models are highly susceptible to overfitting and attention collapse in sparse datasets, often resulting in poor generalization (Bidgoli *et al.* 2026). Also, the integration of pretrained sequence embeddings with tree-based models circumvents the need for excessive parameterization, thereby ensuring more stable and accurate predictions (Kroll *et al.* 2023, 2024).

All machine learning models exhibited reduced performance on the mouse dataset, with MCC values decreasing by approximately 0.2. This reduction likely reflects dataset bias and distributional shift between the human and mouse benchmark sets. As illustrated in Figs. S1 and S2, the mouse kinases have different conformational distributions and low sequence similarity. The majority of the sequence pairs (71.62%) demonstrate an identity percentage of approximately 20%–30%. It is also notable that 92.53% of compound pairs demonstrate a Tanimoto coefficient ranging from zero to 0.2. In contrast, intra-species sequence identity distributions (Fig. S2C and D) exhibited clear bimodal patterns in both datasets, with many sequence pairs showing near-complete identity. This sharp contrast between low inter-species similarity and high intra-species redundancy indicates a substantial distributional shift between the human and mouse benchmark sets, which may encourage models to overfit species-specific patterns and hinder cross-species generalization.

To evaluate the suitability of our model for drug-binding conformation prediction, we first compared the overall performance of individual and stacking models on the human test set. Among the individual models, XGBoost achieved the highest MCC and was therefore selected as the representative single-model baseline. Among the stacking models, the combination of XGBoost and LightGBM yielded the highest MCC. However, because MCC alone may not fully capture the balance of predictions across micro-state classes, we further examined macro-averaged precision, recall, and F1-score. In this comparison, the LightGBM–RF stacking model showed only a marginal decrease in MCC on the human test set compared with XGBoost, while improving macro precision and recall and maintaining the same F1-score. These results indicated that the LightGBM–RF stacking model provided a more balanced overall performance despite a negligible reduction in MCC.

We then compared label-wise prediction performance across microstates using the mouse and human datasets, as shown in Fig. 1 and Table S4. In the mouse dataset, the LightGBM–RF stacking model improved the F1-scores for the BLAminus, DFGout, and Unassigned classes, whereas performance decreased for BLBminus, BLBplus, and DFGother. In the human test set, the same model showed improved performance for BLAplus, BLBminus, BLBtrans, and Unassigned, but reduced performance for ABAminus, BLBplus, DFGout, and DFGother. Notably, the direction of class-wise changes was not always consistent between species, suggesting that micro-state-specific patterns learned from one species may not be directly transferable to another. Considering its balanced macro-level performance on the human test set, its competitive MCC relative to XGBoost, and its highest MCC on the mouse set, the LightGBM–RF stacking model was selected as the final model for KASSPer and used in subsequent benchmark evaluations.

### 3.2 Benchmarking KASSPer prediction for cognate docking

In order to examine the potential of KASSPer prediction to inform the optimal structure for SBVS, a cognate docking experiment was conducted on 24 DUD-E kinases. The pairs of kinase sequence and the SMILES string of the reference structures in the DUD-E set were utilized as inputs for KASSPer. The ligand was docked to the KASSPer predicted state. The benchmark result was compared with ensemble docking using MSM models and Boltz-2 co-folding predictions. For MSM, the compound was docked to all predicted model ensembles, and the conformation with the lowest predicted binding affinity was selected for analysis. For Boltz-2, a kinase sequence and a SMILES string of ligand to be complexed were given as inputs. Table 2 summarizes the results, and the individual results are given in Table S5.

**Table 2:** Cognate docking results of 24 kinases using KASSPer prediction, ensemble docking and co-folding using Boltz-2.

Method	Conformation prediction success (%)	Average RMSD (Å)	Docking success (%)
KASSPer	91.7	3.46	58.3
Ensemble docking	41.7	4.40	33.3
Boltz-2	87.5	1.26	91.7

For ensemble docking, the kinase conformation with the lowest binding affinity was selected for evaluation. The docking success is defined as ligand RMSD less than or equal to 2 Å.

KASSPer demonstrated a high degree of accuracy in predicting the conformational state of the reference complex structure (91.7%), while the docking score proved ineffective in guiding the correct kinase conformational state, with a success rate of 41.7%. In a similar vein, the RMSD and the success rate of the KASSPer prediction are both better than those of the ensemble docking method. Accurate prediction of the receptor conformational state had a significant impact on docking performance. In particular, it improved the accuracy of predicted binding poses. The two KASSPer cases that did not achieve state prediction success were EGFR and IGF1R, with RMSD of 8.43 Å and 7.02 Å, respectively. In the context of ensemble docking, the 10 cases that exhibited the correct conformational state demonstrated an average RMSD of 2.49 Å, while the cases that did not meet this standard exhibited an average RMSD of 5.76 Å, implying that finding a proper protein conformation is important for predicting docking pose precisely.

**Figure 2:**
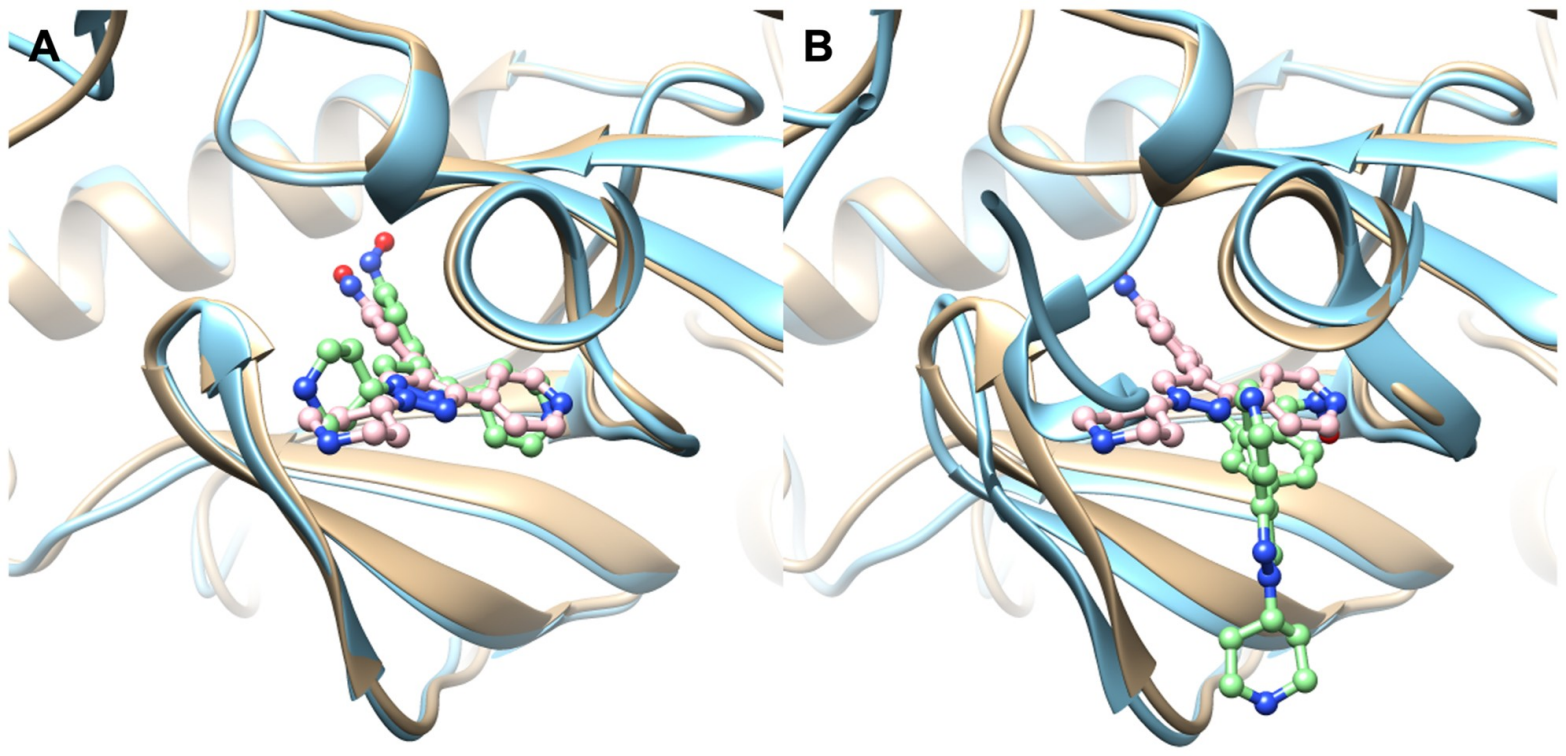
Predicted poses of BRAF cognate docking experiment. Reference structure (PDB ID: 3D4Q) is superimposed on the predicted structures. The cognate ligand docked to (A) KASSPer predicted structure, DFGin_BLAminus, and (B) the lowest AutoDock score, DFGout_BBAminus. The crystal and predicted protein structures are colored gold and sky blue, respectively, while the ligands have pink and green colors.

BRAF serves as an example, illustrating the success of KASSPer prediction to obtain the conformational state and docking pose. In contrast, ensemble docking demonstrated a failure to predict both. The reference complex structure has been classified as DFGin_BLAminus state by KinCoRe. KASSPer’s prediction of the conformational state was accurate, and the docking pose exhibited an RMSD of 1.384 Å (Fig. 2A). However, the lowest AutoDock score was obtained for DFGout conformation, yielding an RMSD of 7.260 Å (Fig. 2B).

A comparison between KASSPer and Boltz-2 highlights differences in intended use and suggests potential complementary use cases. KASSPer slightly outperformed Boltz-2 in conformational state prediction (91.7% vs. 87.5%), while Boltz-2 produced more accurate binding poses with an average RMSD of 1.26 Å and a docking success rate of 91.7%, compared with 3.46 Å RMSD and 58.3% success rate for KASSPer. However, this difference in pose accuracy should be evaluated within the framework of the underlying design of each method. Boltz-2 is an end-to-end co-folding method that jointly generates the receptor conformation and the ligand binding pose, with ligand placement learned directly from large collections of protein-ligand complexes. Conversely, the function of KASSPer is constrained to the selection of the most probable kinase conformation. The placement of the ligand in the docking benchmark was executed by an external docking program, AutoDock-GPU. The pose accuracy of the KASSPer pipeline is therefore constrained by the scoring function and search algorithm of the docking program. Future work should test whether coupling KASSPer with more rigorous docking or rescoring methods, such as AK-Score2 (Hong *et al.* 2024), can narrow this gap.

To further examine this potential state-aware prioritization role, we increased the number of predicted models by Boltz-2 to 50 for AKT1, the protein for which the co-folding method failed to generate a conformation identical to the native structure. The RMSD of the co-folded structure ligand was 0.64. KASSPer successfully predicted the bound state of the complex as the BLAminus state. Among 50 Boltz-2 samples, 9 were classified as BLAminus, 40 as Unassigned, and 1 as BABtrans. The BLAminus group had the lowest ligand RMSD among the state groups (0.33 Å), compared with Unassigned (0.35 Å) and BABtrans (0.56 Å), indicating that the KASSPer-predicted state contained the most accurate Boltz-2 ligand pose in this case. This might imply that the KASSPer and co-folding method, such as Boltz-2, could have a synergy for predicting the complex structure more precisely.

Overall, these results indicate that accurate prediction of kinase conformational state is associated with improved docking pose prediction. By providing a reasonable estimate of the ligand-competent receptor conformation, KASSPer may help reduce uncertainty in docking calculations. This observation indicates that KASSPer predictions could be useful as an initial receptor model in SBVS of kinases with substantial conformational variability.

### 3.3 Applying KASSPer to SBVS

As the next step, we utilized KASSPer prediction for kinase SBVS. For KASSPer screening, the compounds were docked to the KASSPer-predicted conformation and ranked by AutoDock scores. On the other hand, ensemble screening was performed by docking the molecules to all kinase conformations generated by MSM. The lowest predicted binding affinity was selected as a representative score for ranking the compound. A comparison of the two methods is presented in Table 3, and detailed results for individual targets are also provided in Table S6.

**Table 3:** Virtual screening benchmarks of kinases using KASSPer prediction and ensemble screening.

Average dissimilarity	Number of Targets	KASSPer				Ensemble Screening			
		EF1%	EF5%	EF10%	AUC	EF1%	EF5%	EF10%	AUC
All Targets	25	8.70	4.04	2.93	0.67	7.19	3.53	2.62	0.65
0.73–1.00	10	6.85	3.48	2.72	0.67	6.40	3.27	2.42	0.66
0.69–0.72	7	6.93	3.48	2.48	0.66	4.86	3.01	2.36	0.65
0.00–0.68	8	12.56	5.24	3.58	0.67	10.47	4.36	3.10	0.65

The targets are classified by the average dissimilarity of active compounds.

On average, KASSPer screening exhibited superior performance in all metrics when compared with ensemble screening. For EF1%, KASSPer (8.70) exhibited an 21.0% improvement over the ensemble method (7.19). The statistical analysis was conducted using the paired t-test, yielding *P*value less than 0.05 (0.018). Similarly, KASSPer exhibited enhancements in both EF5% (14.4%) and EF10% (11.8%), demonstrating statistical significance with *P*value of 0.014 and 0.034 for EF5% and EF10%, respectively (both < 0.05).

The utilization of multiple conformations of the target protein in the ensemble screening technique is advantageous for the identification of diverse hit molecules. Song *et al.* (2024) calculated the dissimilarity of active compounds of the DUD-E kinase subset using RDKit and found that the ensemble screening using MSM models outperformed the single conformation screening using AF2 and AF3 structures, especially for the targets with high dissimilarity. To investigate whether a screening against the conformer with KASSPer prediction still works for diverse active compounds, we classified the targets into three groups by the average dissimilarity of active compounds (Table 3). Across all target classes and metrics, KASSPer demonstrated superior performance in comparison to the ensemble screening.

**Figure 3:**
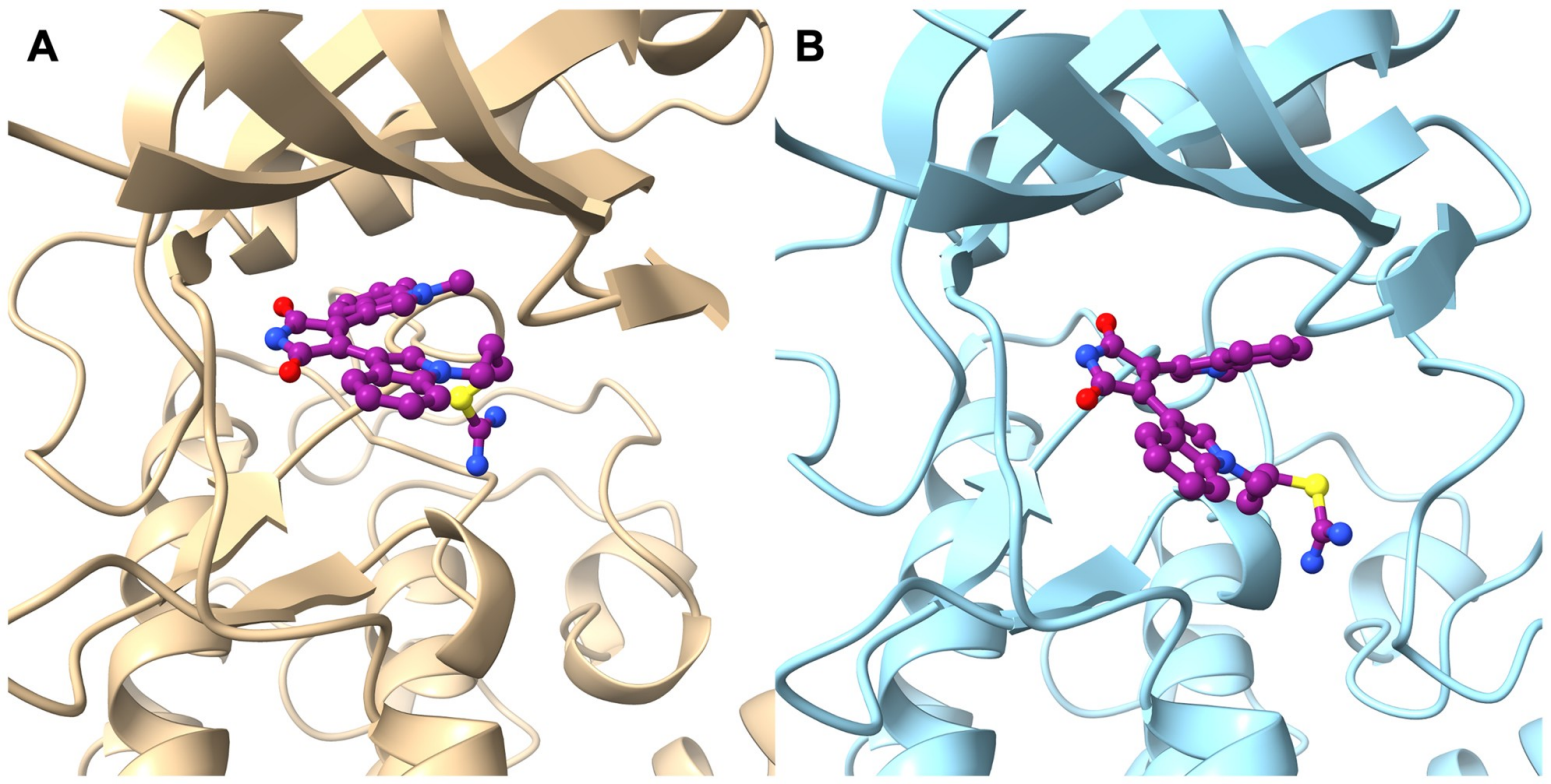
Predicted binding poses of CHEMBL6291, an active compound of PRKCB, to (A) KASSPer predicted structure, DFGin_BLAminus (gold), and (B) the Unassigned (sky blue) with the lowest AutoDock score. The docked ligand is colored purple in both panels.

One of the examples that KASSPer-based SBVS outperformed ensemble screening is PRKCB. The EF1% of KASSPer screening and ensemble screening are 21.55 and 12.63, respectively. Figure 3 shows the docking poses of one of the active compounds, CHEMBL6291. KASSPer predicted the compound binds to DFGin_BLAminus, resulting in a docking score of −11.31 kcal/mol (Fig. 3A). The KASSPer-based screening ranked the compound within the top 1% (38th) out of 8827 compounds. On the other hand, by the ensemble screening, the lowest binding energy was obtained from the Unassigned_Unassigned conformation (−11.45 kcal/mol, Fig. 3B). Although the ensemble screening found the lower energy conformation, it did not rank the molecule within the top 1% (111th).

Although KASSPer is primarily designed to predict the optimal receptor conformation for a given ligand, we also examined how it handles non-binders (decoys) in virtual screening by analysing the 9-dimensional probability vectors assigned across the nine structural states and calculating Silhouette scores in this probability space (Table S7). The results showed strong target dependence, with high positive scores for EGFR (0.557) and WEE1 (0.482) indicating that actives and decoys occupy distinct conformational probability spaces and suggesting that KASSPer can recognize the structural mismatch of non-binders and assign them different conformational preferences. In contrast, negative scores for LCK (−0.504) and IGF1R (−0.401) reflected substantial overlap, indicating that this implicit discrimination depends on the target’s specific chemical and structural space.

In summary, these results suggest that KASSPer-based virtual screening can achieve better performance than ensemble-based screening for kinases. Across the DUD-E kinase subset, screening against a single KASSPer-predicted conformation yielded consistently higher enrichment metrics than ensemble screening, including for targets with high ligand dissimilarity. These findings support the use of KASSPer for kinase SBVS. The method reduces the number of docking calculations required for ensemble screening while maintaining accuracy.

## 4 Conclusion

A significant challenge in the field of SBVS is the flexibility of receptors, with kinases serving as a typical example. These enzymes exhibit a high degree of conformational flexibility at their active site, depending on the bound ligand. While the ensemble screening may offer a solution to this issue, it should be noted that the number of computations increases in proportion to the number of conformations. To address this issue, we developed KASSPer, a language model-based framework to predict kinase conformation when a compound binds. A stacking model of LightGBM and RF was selected based on the validation result. The program demonstrated a high degree of accuracy in predicting the kinase conformation for the cognate docking benchmark, thereby facilitating the precise prediction of docking poses. In the context of the SBVS benchmark, KASSPer demonstrated superior performance in comparison to ensemble screening, as indicated by EF1%, EF5%, EF10%, and AUC. The findings indicate that KASSPer can capture ligand-associated kinase state preferences and may provide a computationally efficient alternative to exhaustive ensemble docking in selected SBVS settings.

Despite strong screening performance, the embeddings used by KASSPer are not directly interpretable, which hinders biological insight into sequence contributions to DFG state selection. Future work will focus on designing embedding strategies with built-in feature attribution to reveal which protein residues and ligand substructures most strongly influence state predictions. Such insights will enhance model interpretability and guide rational design of kinase modulators. Overall, KASSPer provides a practical framework for incorporating ligand-specific kinase conformational-state prediction into SBVS workflows.

## Supplementary Material

btag481_Supplementary_Data

## Data Availability

The receptor structures predicted by the MSM protocol are available at https://zenodo.org/records/8272608. DUD-E benchmark compound library is available at dude.docking.org.
