# Supplementary material for: KASSPer: kinase active site structure prediction using protein and ligand language models and its application to virtual screening
Source: Bioinformatics. 2026 Jul 1;42(7):btag481. doi: 10.1093/bioinformatics/btag481 (PMC13384051; doi:10.1093/bioinformatics/btag481)

Supplementary Data for

KASSPer: Kinase Active Site Structure Prediction using Protein and Ligand Language Models and Its Application to Virtual Screening

Wonkyeong Jang[1] and Woong-Hee Shin[1,2]*

1 Department of Biomedical Informatics, Korea University College of Medicine, Seoul 02708, Republic of Korea

2. Arontier, Co. Seoul 06735, Republic of Korea

Contact: Woong-Hee Shin (whshin@korea.ac.kr)

Table S1. Hyperparameters for the machine learning models benchmarked.

Models	Name	Parameters
XGBoost	n_estimators	50, 100, 200
	max_depth	3, 5, 7
	learning_rate	0.01, 0.1, 0.2
	colsample_bytree	0.7, 1.0
LightGBM	n_estimators	50, 100, 200
	max_depth	3, 5, 7
	learning_rate	0.01, 0.1, 0.2
	bagging_fraction	0.7, 1.0
	feature_fraction	0.7, 1.0
CatBoost	tuned iterations	50, 100, 200
	max_depth	3, 5, 7
	learning_rate	0.01, 0.1, 0.2
	subsample	0.7, 1.0
	rsm	0.7, 1.0
Random Forest	n_estimators	50, 100, 200
	max_depth	None, 10, 20
	max_features	sqrt, log2
	min_samples_split	2, 5
Logistic Regression	hyperparameter C	0.01, 0.1, 1, 10
	max_iter	100, 200
	multi_class	ovr, multinomial
	class_weight	None, balanced
SVM	hyperparameter C	0.01, 0.1, 1, 10
	kernel function	linear, rbf
	gamma	scale, auto
	max_iter	100, 200

Table S2. Performance of individual models.

Sets	Single Model								Stacking Ensemble		
	**XGBoost**	**LightGBM**	**CatBoost**	**Random Forest**	**Logistic Regression**	**SVM**	**Cross Attention**	**Concatenate**	**XGBoost + LightGBM**	**LightGBM + Random Forest**	**XGBoost + Random Forest**
					Human						
MCC	0.75	0.74	0.71	0.72	0.65	0.45	0.69	0.68	0.75	0.74	0.74
Precision	0.71	0.73	0.70	0.70	0.63	0.48	0.65	0.64	0.74	0.75	0.73
Recall	0.66	0.66	0.63	0.64	0.59	0.47	0.63	0.63	0.66	0.67	0.65
F1-Score	0.68	0.68	0.65	0.66	0.6	0.47	0.63	0.62	0.68	0.68	0.67
					Mouse						
MCC	0.52	0.51	0.43	0.49	0.38	0.25	0.45	0.39	0.51	0.53	0.53
Precision	0.38	0.37	0.31	0.36	0.25	0.25	0.35	0.30	0.38	0.39	0.36
Recall	0.37	0.36	0.31	0.33	0.24	0.29	0.35	0.33	0.34	0.36	0.32
F1-Score	0.35	0.32	0.28	0.30	0.22	0.24	0.32	0.29	0.32	0.34	0.31

Table S3. Performance of individual models with reduced embedding dimensions using PCA.

Sets	Single Model								Stacking Ensemble		
	**XGBoost**	**LightGBM**	**CatBoost**	**Random Forest**	**Logistic Regression**	**SVM**	**Cross Attention**	**Concatenate**	**XGBoost + LightGBM**	**LightGBM + Random Forest**	**XGBoost + Random Forest**
Human											
MCC	0.75	0.74	0.71	0.63	0.63	0.47	0.70	0.67	0.74	0.73	0.73
Precision	0.70	0.71	0.73	0.81	0.61	0.50	0.65	0.64	0.73	0.71	0.70
Recall	0.63	0.65	0.63	0.49	0.57	0.48	0.64	0.63	0.66	0.65	0.64
F1-Score	0.65	0.67	0.65	0.46	0.58	0.48	0.64	0.62	0.68	0.66	0.66

Table S4. Detailed results of XGBoost and a stacking model of LightGBM with Random Forest for the individual class. For the mouse set, no crystal structures were assigned as BLBtrans.

Conformational State	Human		Mouse	
	**XGBoost**	**LightGBM + RF**	**XGBoost**	**LightGBM + RF**
		**Precision**		
ABAminus	0.88	0.84	0.00	0.00
BLAminus	0.86	0.87	0.72	0.78
BLAplus	0.86	0.92	0.00	0.00
BLBminus	0.55	0.91	0.90	0.91
BLBplus	0.81	0.77	0.35	0.30
BLBtrans	0.57	0.58	-	-
Unassigned	0.7	0.71	0.18	0.25
DFGout	0.35	0.38	0.75	0.81
DFGothers	0.82	0.78	0.47	0.43
		**Recall**		
ABAminus	0.81	0.78	0.00	0.00
BLAminus	0.96	0.95	0.88	0.89
BLAplus	0.67	0.67	0.00	0.00
BLBminus	0.37	0.33	0.30	0.16
BLBplus	0.79	0.80	0.49	0.56
BLBtrans	0.71	0.88	-	-
Unassigned	0.42	0.44	0.11	0.11
DFGout	0.38	0.31	0.88	0.96
DFGothers	0.83	0.84	0.70	0.60
		**F1-Score**		
ABAminus	0.84	0.81	0.00	0.00
BLAminus	0.91	0.91	0.79	0.83
BLAplus	0.75	0.77	0.00	0.00
BLBminus	0.44	0.49	0.45	0.27
BLBplus	0.80	0.78	0.41	0.39
BLBtrans	0.63	0.70	-	-
Unassigned	0.53	0.54	0.13	0.15
DFGout	0.82	0.81	0.81	0.88
DFGothers	0.36	0.34	0.56	0.50

Table S5. Individual result of the cognate docking experiment. The structural states of the ensemble docking were selected based on the lowest AutoDock-GPU score.

Protein	Conformational State	KASSPer		Ensemble Docking		Boltz2	
		Predicted State	RMSD (Å)	Lowest Scoring State	RMSD (Å)	Predicted State	RMSD (Å)
ABL1	DFGout	DFGout	0.29	DFGout	0.29	DFGout	0.68
AKT1	BLAminus	BLAminus	0.57	BLAminus	0.57	Unassigned	0.64
AKT2	BLAminus	BLAminus	0.72	BLAminus	0.72	BLAminus	0.34
BRAF	BLAminus	BLAminus	1.38	DFGout	7.26	BLAminus	0.98
CDK2	BLBtrans	BLBtrans	9.65	DFGout	11.37	BLBtrans	0.94
CSF1R	DFGout	DFGout	8.21	BLBtrans	7.82	DFGout	1.03
EGFR	BLBplus	BLAminus	8.43	BLBtrans	10.03	BLBplus	1.12
FGFR1	BLAplus	BLAplus	0.35	DFGout	0.89	BLAplus	0.39
IGF1R	DFGothers	Unassigned	7.02	DFGout	6.90	Unassigned	1.48
JAK2	ABAminus	ABAminus	1.32	DFGout	6.92	ABAminus	1.07
KDR	DFGout	DFGout	1.23	DFGout	1.23	DFGout	0.45
LCK	BLAminus	BLAminus	0.73	BLBplus	6.66	BLAminus	0.73
MAP2K1	BLBplus	BLBplus	2.59	BLBplus	2.59	BLBplus	10.61
MAPK1	BLAminus	BLAminus	1.20	DFGothers	3.46	BLAminus	0.44
MAPK10	ABAminus	ABAminus	9.17	BLAplus	8.84	ABAminus	0.44
MAPK14	BLBplus	BLBplus	6.71	BLAminus	2.59	DFGout	4.10
MAPKAPK2	BLAminus	BLAminus	0.82	BLBplus	1.54	BLAminus	0.79
MET	DFGout	DFGout	11.33	DFGout	11.33	DFGout	0.52
PLK1	BLAminus	BLAminus	1.86	Unassigned	3.28	BLAminus	0.99
PRKCB	BLAminus	BLAminus	4.35	BLAminus	4.35	BLAminus	0.81
PTK2	Unassigned	Unassigned	0.88	Unassigned	0.88	Unassigned	0.44
ROCK1	BLAminus	BLAminus	1.22	DFGout	3.02	BLAminus	0.94
TGFBR1	BLAminus	BLAminus	0.88	BLAminus	0.88	BLAminus	0.18
WEE	BLAminus	BLAminus	2.10	BLAminus	2.10	BLAminus	0.27

Table S6. Virtual screening benchmark result of KASSPer and ensemble docking for DUD-E kinase subset.

Kinase	Average Dissimilarity	KASSPer				Ensemble Screening			
		EF1%	EF5%	EF10%	AUC	EF1%	EF5%	EF10%	AUC
ABL1	0.73	10.47	4.62	3.30	0.71	9.36	3.74	2.47	0.64
AKT1	0.68	9.57	5.19	3.92	0.74	12.64	5.46	3.79	0.74
AKT2	0.69	17.12	5.82	3.76	0.77	12.84	5.65	3.93	0.78
BRAF	0.70	11.84	4.87	3.16	0.74	7.23	5.26	3.22	0.71
CDK2	0.78	2.53	1.65	1.48	0.61	5.70	2.57	2.19	0.66
CSF1R	0.73	6.03	3.13	2.71	0.63	5.43	3.50	2.17	0.64
EGFR	0.70	8.29	3.91	2.73	0.64	4.42	3.06	2.21	0.60
FGFR1	0.69	6.50	5.03	3.81	0.70	2.16	2.45	2.16	0.66
IGF1R	0.69	4.75	3.24	2.43	0.71	6.78	3.24	2.97	0.74
JAK2	0.73	5.61	2.43	1.68	0.57	5.61	2.43	1.87	0.58
KDR	0.75	9.06	4.55	3.32	0.70	9.06	4.60	3.40	0.70
KIT	0.73	12.67	5.18	3.92	0.72	12.67	4.09	3.01	0.70
LCK	0.73	6.43	3.19	2.52	0.66	4.28	2.90	2.33	0.62
MAP2K1	0.71	0.00	0.99	0.91	0.58	0.00	0.83	0.83	0.56
MAPK1	0.75	11.46	5.58	3.80	0.74	6.37	4.82	3.67	0.72
MAPK10	0.58	2.89	2.31	2.50	0.66	3.85	2.89	2.31	0.65
MAPK14	0.75	2.25	1.90	1.71	0.63	3.46	2.49	1.90	0.64
MAPKAPK2	0.68	5.99	3.77	3.17	0.68	3.99	2.78	2.67	0.67
MET	0.70	0.00	0.48	0.54	0.45	0.60	0.60	1.20	0.52
PLK1	0.65	6.55	4.30	3.37	0.62	1.87	1.50	1.68	0.54
PRKCB	0.56	21.55	7.71	5.04	0.64	12.63	5.49	3.78	0.67
PTK2	0.68	0.00	0.60	0.60	0.46	0.00	0.00	0.40	0.37
ROCK1	0.74	2.00	2.60	2.80	0.72	0.00	1.20	1.10	0.68
TGFBR1	0.64	5.28	4.36	3.01	0.69	5.28	3.60	3.31	0.69
WEE1	0.39	48.64	13.71	7.06	0.89	43.48	13.16	6.86	0.88
Average		8.70	4.04	2.93	0.67	7.19	3.53	2.62	0.65

Table S7. Silhouette Score of individual targets. Nine-dimensional vector that containing the probabilities of structural states is utilized to compute the score. Scores range from -1 to +1. Positive values indicate separation between actives and decoys, near-zero values indicate overlap, and negative values indicate indistinguishable structural preferences.

Target	Silhouette Score
ABL1	-0.118
AKT1	0.276
AKT2	0.212
BRAF	0.107
CDK2	-0.195
CSF1R	-0.324
EGFR	0.557
FGFR1	0.123
IGF1R	-0.401
JAK2	-0.092
KDR	0.128
KIT	-0.069
LCK	-0.504
MAP2K1	-0.063
MAPK1	-0.001
MAPK10	0.374
MAPK14	-0.034
MAPKAPK2	0.174
MET	-0.112
PLK1	0.130
PRKCB	0.312
PTK2	0.107
ROCK1	0.335
TGFBR1	0.022
WEE1	0.482

Figure S1. The class distribution of kinase crystal structures. The y-axis is the percentage of the class.

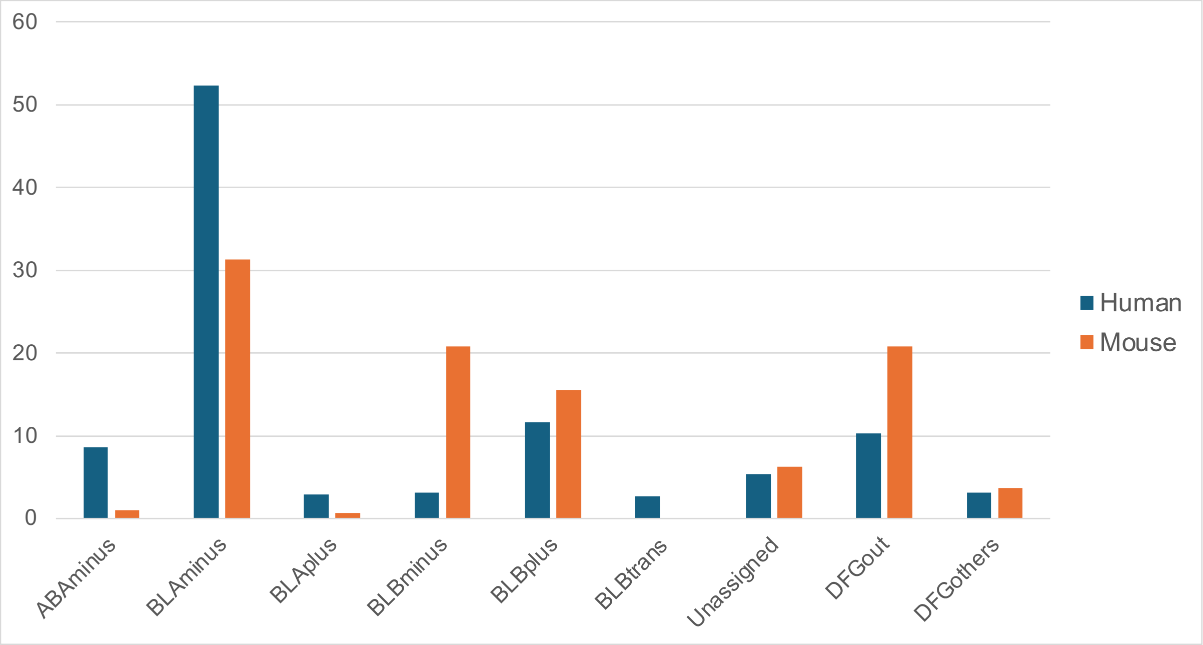

Figure S2. Analysis between the human set and the mouse set. **A, B.** Inter-species sequence identity distribution of proteins (**A**) and Tanimoto similarity distribution of ligands (**B**). **C, D.** Human intra-species sequence identity distribution (**C**) and Mouse intra-species sequence identity distribution (**D**).

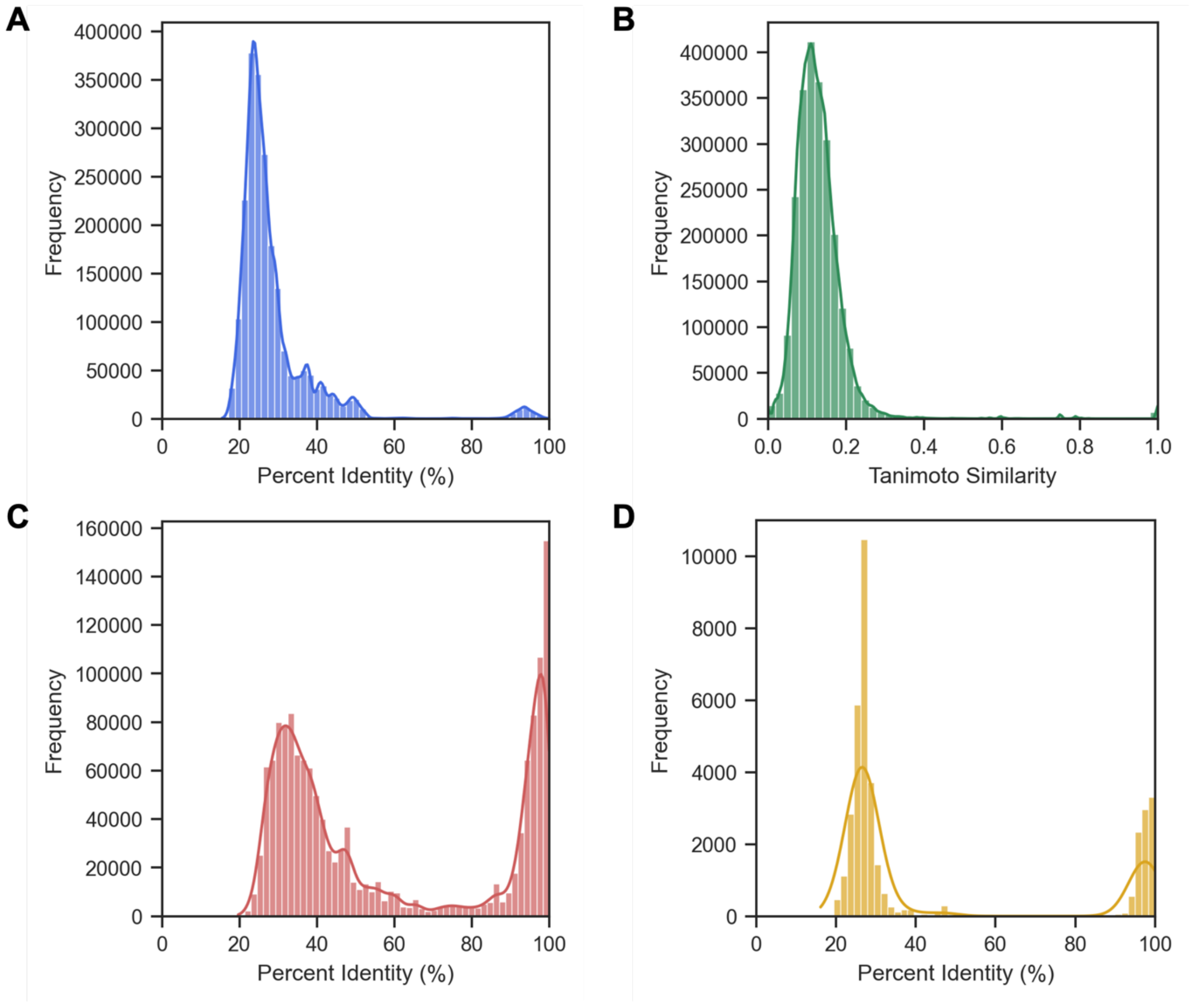

Figure S3. Learning curve analysis for eight models evaluated in this study. Each model was trained using increasing fractions of the training dataset (10–100%) while maintaining class distribution. Performance was evaluated using the Matthews correlation coefficient (MCC). The MCC values of training and test results are represented as a gray dashed line and a blue solid line with dots, respectively.

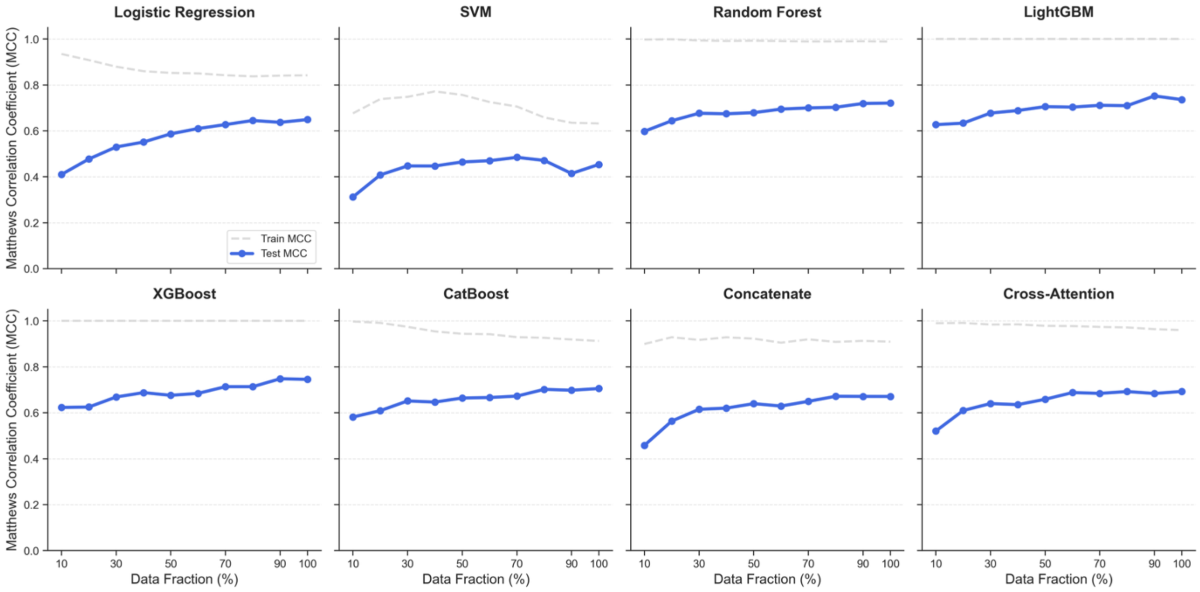

Supplement: btag481_Supplementary_Data [file btag481_supplementary_data.pdf]
